# Effects of LL-37 on Gingival Fibroblasts: A Role in Periodontal Tissue Remodeling?

**DOI:** 10.3390/vaccines6030044

**Published:** 2018-07-23

**Authors:** Maelíosa T. C. McCrudden, Katherine O’Donnell, Chris R. Irwin, Fionnuala T. Lundy

**Affiliations:** 1 Centre for Experimental Medicine, Wellcome-Wolfson Institute for Experimental Medicine, School of Medicine, Dentistry and Biomedical Sciences, Queen’s University Belfast, Belfast BT7 9BL, UK; m.mccrudden@qub.ac.uk; 2 Centre for Dentistry, School of Medicine, Dentistry and Biomedical Sciences, Queen’s University Belfast, Belfast BT12 6BA, UK; kodonnell08@qub.ac.uk (K.O.); c.r.irwin@qub.ac.uk (C.R.I.)

**Keywords:** fibroblast, gingival, growth factors, host defence peptide, LL-37, oral, periodontal, wound repair

## Abstract

Mounting evidence suggests that the host defence peptide, LL-37, plays a role in both inflammation and in wound healing; however, the role of this peptide in the remodeling and maintenance of oral tissues is not yet fully understood. Fibroblasts are the most abundant cell type within the periodontal tissues, and gingival fibroblasts play an important role in maintaining and repairing the gingival tissues which are constantly exposed to external insults. In this study we examined the direct effects of LL-37 treatment on gingival fibroblasts and found that LL-37 significantly increased secretion of both interleukin 8 (IL-8) and IL-6 from these cells. LL-37 tended to decrease matrix metalloproteinase (MMP) activity in gingival fibroblasts, but this decrease did not reach statistical significance. LL-37 significantly increased tissue inhibitor of metalloproteinase-1 (TIMP-1) production by gingival fibroblasts, but had no significant effect on TIMP-2 levels. LL-37 was also shown to significantly increase production of basic fibroblast growth factor (bFGF), hepatocyte growth factor (HGF), and keratinocyte growth factor (KGF) in gingival fibroblasts. Taken together, these results suggest an important role for the host defence peptide, LL-37, in modulating the fibroblast response to remodeling in periodontal tissues.

## 1. Introduction

Human cathelicidin (hCAP18) is an 18 kDa protein [1], derived from the cathelicidin family of host defence peptides, and is the only cathelicidin protein in humans [2,3]. All cathelicidins have an N-terminal signal sequence, with a conserved cathelin-like prodomain and variable C-terminal antimicrobial domain [4]. hCAP18 is synthesised by epithelial cells and keratinocytes and LL-37, the 37-residue C-terminal peptide of hCAP18, is found in a number of tissues and bodily fluids, including saliva, gastric fluid, semen, sweat, plasma, airway surface liquid, and breast milk [5]. Previously, we quantified LL-37 in gingival crevicular fluid (GCF) from periodontally healthy and diseased patients and showed that LL-37 was increased in periodontitis patients [6]. hCAP18 is known to be abundant in the specific granules of neutrophils [7] and is cleaved by proteinase-3 following exocytosis [8]. LL-37 has broad-spectrum antimicrobial activity against Gram-positive and Gram-negative bacteria along with fungi and some viruses [4]. Mice deficient in the murine cathelicidin, CRAMP, exhibited increased risk of necrotising skin infections, supporting the theory that LL-37 has a role in immune defence [3,9]. Neutrophils from patients with morbus Kostmann syndrome, a severe congenital neutropenia, are deficient in hCAP18/LL-37 and these patients suffer from recurrent bacterial infections. They also suffer from severe periodontal disease, suggesting an important role for LL-37 in prevention of infection and resolution of inflammation in oral tissues [10,11]. Interestingly, studies have been carried out to deduce whether endogenous expression of LL-37 in oral tissues can be bolstered, via treatment with vitamin D_3_, so as to boost the innate immune response and prevent colonisation of the gingival epithelium by periodontal pathogens [12]. It has been proposed that LL-37 functions as an immune watchdog at lower concentrations (e.g., 2 μg/mL, equivalent to 0.4 μM), while at higher concentrations (such as those induced by bacterial products), the main role of LL-37 is chemotaxis of immune cells to fight the infection [13]. LL-37 is a chemoattractant for human monocytes, T cells, and mast cells. It induces production of chemokines and is a potent anti-endotoxic agent [5,8,14]. Additional anti-inflammatory effects of LL-37 are thought to be mediated by its ability to reduce lipopolysaccharide (LPS)- and lipotecihoic acid (LTA)-induced stimulation of proinflammatory cytokines [8].

Mounting evidence thus suggests that LL-37 is a component of the innate immune system in humans, playing a role in inflammation as well as in tissue remodeling and wound healing [15,16] Effective tissue remodeling and wound repair relies on several events including epithelial proliferation, migration, and differentiation, with the majority of these processes being controlled by growth factors. Indeed, the low levels of LL-37 detected in chronic ulcers have been implicated in impaired restoration of the epidermis [3]. LL-37 has been detected in GCF from healthy subjects as well as in periodontitis patients [6,17,18]. As a result of the chronic inflammatory process that is the hallmark of periodontal disease, the periodontal pocket epithelium becomes micro-ulcerated, exposing the underlying fibroblasts. Fibroblasts are the most abundant cell type within the periodontal tissues and are responsible for the turnover and repair of the extracellular matrix. Previous studies have determined that, at concentrations of ≥5 μg/mL (equivalent to 1 μM), LL-37 has a range of biological functions including LPS-binding [13] and stimulation of chemokine production by epithelial cells [8]. However, the effects of LL-37 on underlying fibroblast cells have been less well documented and we therefore studied the effects of LL-37 on key mediators of tissue remodeling in gingival fibroblasts. To this end, the production of basic fibroblast growth factor (bFGF), keratinocyte growth factor (KGF), and hepatocyte growth factor (HGF) were determined; in addition, the secretion of pro-inflammatory cytokines (IL-8 and IL-6) following LL-37 treatment of gingival fibroblasts was measured. Furthermore, the effect of peptide treatment on the activities of matrix metalloproteinases (MMP) and reciprocal expression of tissue inhibitors of metalloproteinases (TIMP) was also determined as they have both been shown to have roles in the extracellular matrix remodeling associated with periodontal disease. 

## 2. Materials and Methods

### 2.1. Materials

Synthetic LL-37 (>95% purity) was purchased from Innovagen, Lund, Sweden. All human growth factor Quantikine ELISA kits (HGF, bFGF, KGF), TIMP-1 and TIMP-2 Quantikine ELISA kits, and IL-8 and IL-6 ELISA reagents were purchased from R&D Systems, Abingdon, UK. AnaSpec 520 MMP FRET Substrate XIV was obtained from AnaSpec, Fremont, CA, USA. MTT reagents were purchased from Sigma Aldrich, Dorset, UK. 

### 2.2. Cell Culture and Treatment

Gingival tissue was obtained from healthy donors with ethical approval (08/NIR03/15). Cell lines were obtained by explant culture, as previously described [19]. Fibroblasts were maintained in Dulbecco’s Minimal Essential Medium (DMEM; Invitrogen, Paisley, UK) supplemented with 10% heat-inactivated foetal bovine serum (FBS; Invitrogen, Paisley, UK), 2 mM L-glutamine (Invitrogen, Paisley, UK), and 1% (*v*/*v*) penicillin/streptomycin (PAA Laboratories GmbH, Pasching, Austria). Cells were maintained at 37 °C in a humidified atmosphere of 5% CO_2_. Fibroblasts were grown to confluence before being harvested by trypsinisation and subcultured. 

### 2.3. ELISA Protocols

Gingival fibroblasts were seeded into the wells of a 24-well culture plate at a density of 1 × 10^5^ cells/well and cultured until confluent. Following incubation for 24 h in DMEM containing 1% (*v*/*v*) FBS, to induce quiescence, cells were treated with LL-37 at concentrations of 0.2, 1, or 2 μM or in medium containing the vehicle (control). After incubation, the cell supernatants were collected and stored at −20 °C until required. The concentrations of IL-8 or IL-6 in the cell-free supernatants were measured by ELISA using DuoSet ELISA development kits from R&D Systems (Abingdon, UK); bFGF, HGF, and KGF concentrations were quantified using the commercially available human growth factor Quantikine ELISA kits (R&D Systems, Abingdon, UK) and TIMP-1 and TIMP-2 concentrations were determined using the Human TIMP-1 and TIMP-2 Quantikine ELISA kits (R&D Systems, Abingdon, UK), all according to manufacturer’s instructions. Absorbance was determined at 405 nm on a Tecan Genios microtitre plate reader (Tecan, Reading, UK) using Magellan software (Tecan, Reading, UK).

### 2.4. MMP Activity

Total MMP activity profiles of all individual cell supernatants were determined using the AnaSpec 520 MMP FRET Substrate XIV (AnaSpec, Fremont, CA, USA), using a method previously described by our group [20]. This substrate is cleaved by MMP-1, -2, -3, -7, -8, -9, -12, and -13 and as such, was determined to be an ideal substrate for use in the determination of total MMP profiles of samples. Prior to use in activity assays, the substrate was reconstituted to a concentration of 1 mM in dimethyl sulfoxide (DMSO) and subsequently diluted to 100 μM in MMP Assay Buffer (AnaSpec, Fremont, CA, USA). The substrate was then aliquoted and stored at −20 °C until required. Before use, the substrate was diluted to 10 μM in MMP Assay Buffer and then 50 μL aliquots of this solution, along with 50 μL of the respective samples (diluted 1/10), were added to the appropriate wells of a Greiner 96-well black plate (Sigma Aldrich, Dorset, UK). Fluorescence measurements were immediately recorded at excitation and emission wavelengths of 485 nm and 525 nm, respectively. Measurements were recorded every 5 min for a duration of 70 min on a Tecan Genios microtitre plate reader (Tecan, Reading, UK) using Magellan software. The MMP activity profiles of the samples were quoted as relative fluorescence units (RFU) per minute (RFU/min). Treatment with 10 mM EDTA for 10 min prior to addition of substrate was included as an additional control to confirm that the substrate was MMP specific. 

### 2.5. MTT Assay of LL-37-Treated Gingival Fibroblast Cells

Cell viability assays were carried out as described previously [21]. Briefly, gingival fibroblasts were seeded into a 96-well culture plate at 1 × 10^4^ cells/well and maintained until they reached confluence. The medium was then replaced with DMEM containing 1% (*v*/*v*) FBS so as to induce quiescence overnight. LL-37 stocks were diluted in DMEM to final concentrations of 0.2, 1, or 2 μM for cell treatments. Vehicle controls were included in all experiments. Six replicates of each cell treatment were carried out. Cells were incubated at 37 °C for 24 h, before 10 μL of thiazolyl blue tetrasodium bromide (MTT) reagent (Sigma-Aldrich, Dorset, UK) was added to each well and incubated for 2 h at 37 °C. Cell culture media were removed, the plate was air dried, and 200 μL dimethyl sulfoxide (DMSO) (Sigma Aldrich, Dorset, UK) was then added to each well and the absorbance determined at 405 nm using a Tecan Genios microtitre plate reader (Tecan, Reading, UK) and Magellan software.

### 2.6. Statistical Analyses

All statistical analyses were performed using GraphPad PRISM 4.0 software package (San Diego, CA, USA). Cells incubated in medium containing the vehicle only served as controls in all experiments. All data were analysed by one-way analysis of variance (ANOVA) followed by Bonferroni’s multiple comparison test. A *p*-value of < 0.05 was considered statistically significant.

## 3. Results

### 3.1. Cytokine Secretion by Gingival Fibroblasts Following Treatment with Exogenous LL-37

**Figure 1:**
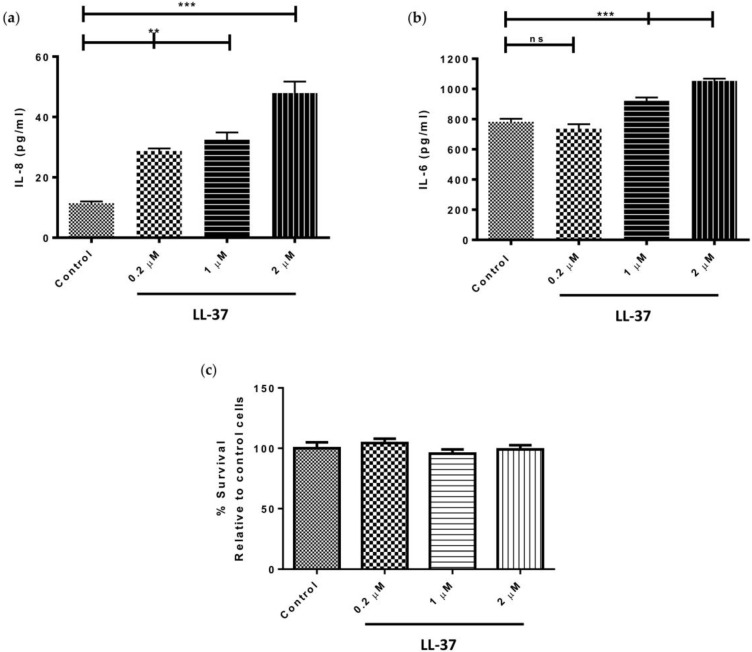
Direct effects of LL-37 on secretion of the pro-inflammatory cytokines (**a**) interleukin (IL)-8 and (**b**) IL-6 by gingival fibroblasts. Cells were incubated in medium containing the vehicle only (Control) or vehicle and peptide (0.2, 1, or 2 μM), (IL-8 mean ± SD, *n* = 3; IL-6 mean ± SD, *n* = 6); (**c**) The percentage cell survival of gingival fibroblasts, relative to that of control, following a 24 h exposure to 0.2, 1, or 2 μM LL-37. Cells were incubated in medium containing the vehicle only (Control) or vehicle and peptide (0.2, 1, or 2 μM), (mean ± SD, *n* = 6). Data were analysed by one-way analysis of variance followed by Bonferroni’s multiple comparison test (ns = nonsignificant; ** = *p* < 0.01; *** = *p* < 0.001). For cell survival experiments, all comparisons were nonsignificant.

Treatment of gingival fibroblasts with 0.2, 1, or 2 μM LL-37, significantly upregulated the levels of IL-8 produced by the cells in a dose-dependent fashion (*p* < 0.05) (Figure 1a). Specifically, IL-8 concentrations reached a maximum of 47.9 ± 6.6 pg/mL following treatment with 2 μM LL-37. In the case of IL-6 secretion, however, only the higher concentrations of LL-37 (1 and 2 μM) significantly upregulated IL-6 production (*p* < 0.05) (Figure 1b). Gingival fibroblast viability was shown to be unaffected by treatment with exogenous LL-37 at concentrations of 0.2, 1, or 2 μM (Figure 1c).

### 3.2. Effect of LL-37 on MMP Activity and TIMP Expression by Gingival Fibroblasts

**Figure 2:**
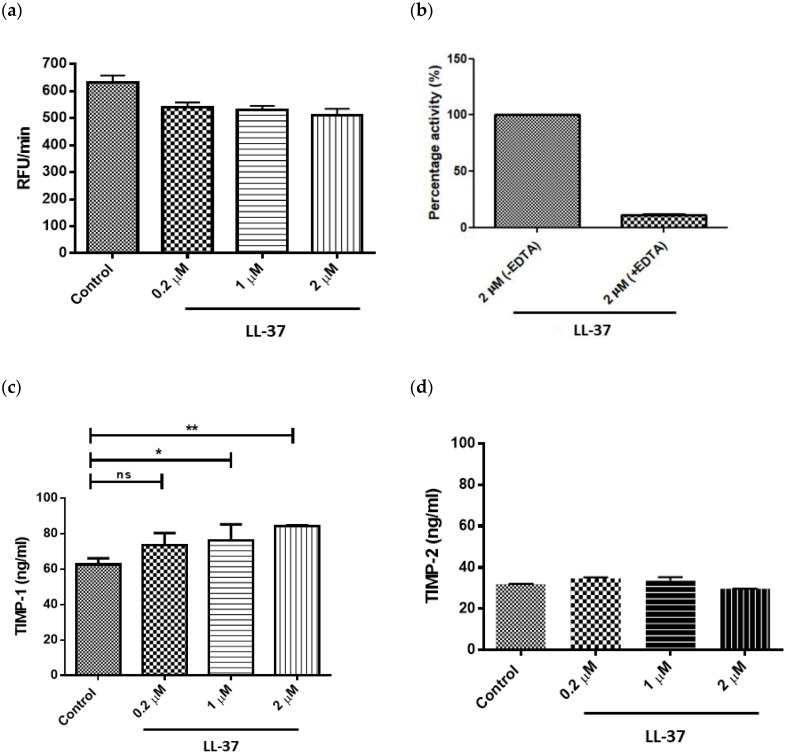
Direct effects of LL-37 on matrix metalloproteinase (MMP) activity and tissue inhibitor of metalloproteinase (TIMP) production. Effects of LL-37 on (**a**) secreted MMP activities and (**b**) inhibition of MMP activities in the presence of EDTA in LL-37-treated gingival fibroblasts. Effects of LL-37 on (**c**) TIMP-1 and (**d**) TIMP-2 secretion by gingival fibroblasts. Cells were incubated in medium containing the vehicle only (Control) or vehicle and peptide (0.2, 1, or 2 μM). MMP activities were expressed in relative fluorescent units (RFU) per minute, RFU/min, mean ± SD, *n* = 6; inhibition assays were expressed as % of substrate turnover in absence of the inhibitor EDTA, *n* = 3; TIMP-1 mean ± SD, *n* = 4; TIMP-2 mean ± SD, *n* = 4). Data were analysed by one-way analysis of variance followed by Bonferroni’s multiple comparison test (ns = nonsignificant; * *p* < 0.05; ** = *p* < 0.01). EDTA inhibition studies were not subject to statistical analysis; EDTA treatment was shown to reduce MMP activity by approximately 90%. In the comparison of MMP activities and TIMP-2 concentrations, all were found to be nonsignificant.

Treatment of gingival fibroblasts with LL-37 at concentrations of 0.2, 1, or 2 μM led to a decrease in total MMP activity; however, these reductions were not statistically significant at any of the concentrations tested (Figure 2a). As expected, MMP treatment with 10 mM EDTA for 10 min prior to addition of substrate confirmed that the substrate (AnaSpec 520 MMP FRET Substrate XIV) was MMP specific (Figure 2b). LL-37 upregulated TIMP-1 secretion by gingival fibroblasts in a dose-dependent manner, with statistically significant increases in TIMP-1 concentrations following addition of LL-37 at 1 and 2 μM (Figure 2c). In contrast, LL-37 had no effect on TIMP-2 secretion at any of the peptide concentrations tested (Figure 2d).

### 3.3. Growth Factor Secretion by Gingival Fibroblasts Following Incubation with LL-37

**Figure 3:**
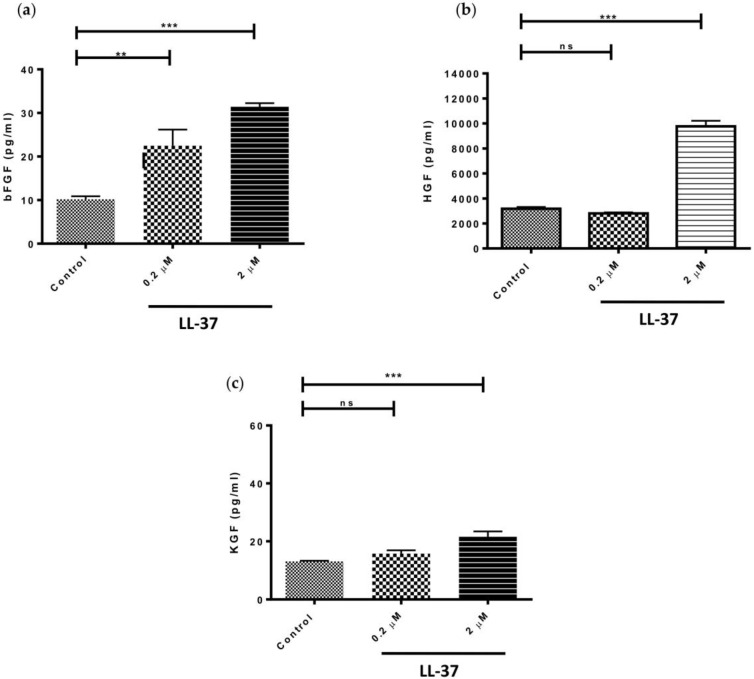
Direct effects of LL-37 on secretion of various growth factors (**a**) bFGF, (**b**) HGF, or (**c**) KGF by gingival fibroblasts. Cells were incubated in medium containing the vehicle only (Control) or vehicle and peptide (0.2 or 2 μM) (bFGF mean ± SD, *n* = 6; HGF mean ± SD, *n* = 6; KGF mean ± SD, *n* = 6). Data were analysed by one-way analysis of variance followed by Bonferroni’s multiple comparison test (ns = nonsignificant; ** = *p* < 0.01; *** = *p* < 0.001).

Based on the adage that LL-37, at concentrations ≥5 μg/mL (equivalent to ≥1 μM), has a range of biological functions [8,19] and the fact that concentrations above this exhibited the greatest effect on pro-inflammatory cytokine and TIMP-1 production by gingival fibroblasts, we investigated the effects of LL-37 on growth factor expression at concentrations of 0.2 and 2 μM. LL-37 significantly upregulated the production of bFGF by fibroblasts at both concentrations tested (Figure 3a). In contrast, production of HGF and KGF by the cells was only significantly increased following treatment with 2 μM peptide (Figure 3b,c). Interestingly, addition of LL-37 at a concentration of 2 μM led to threefold increases in both bFGF and HGF production but only a 1.6-fold increase in the case of KGF. 

## 4. Discussion

The importance of LL-37 in prevention of infection and resolution of inflammation in oral tissues is demonstrated in patients with morbus Kostmann syndrome who suffer from recurrent, severe periodontitis [10]. LL-37 is a multifunctional molecule and has been shown to exhibit a wide range of immunomodulatory functions beyond antimicrobial activity [22,23]. The effects of host defence peptides, such as LL-37, on oral fibroblasts have not been studied extensively, despite the fact that these cells have key roles in healing and repair. One previous study had shown that LL-37 upregulated IL-8 in gingival fibroblasts [24], prompting us to further examine the response of gingival fibroblasts to LL-37 treatment. We demonstrated that LL-37 can stimulate the production of IL-8 and IL-6 by gingival fibroblasts. The significantly increased production of IL-8 by gingival fibroblasts is in accordance with previous work which demonstrated that LL-37 induced IL-8 release by lung epithelial cell lines [11] and gingival fibroblasts [24]. The mechanism by which LL-37 induces IL-8 production has also been reported and is known to involve the P2X7 receptor and the MEK1/2-dependent p44/42 MAP kinases [24]. It has also been shown that LL-37 can directly activate the P2X7 receptor by a mechanism that is independent of the release of endogenous ATP [25]. IL-8 is a neutrophil chemoattractant and, as such, plays an important role in mediating the elimination of bacteria from the gingival crevice via the direct antimicrobial action of neutrophil-derived LL-37. We also showed that LL-37 had a significant effect on IL-6 secretion by gingival fibroblasts, but only at concentrations ≥1 μM. The lack of effect observed at low LL-37 concentrations may be due, in part, to the higher basal levels of IL-6 secreted by fibroblasts. An effect on the induction of IL-6 by bronchial epithelial cells has previously been reported but only following treatment at high concentrations of LL-37 (>4 μM) [26]. Further studies on the effects of LL-37 on the production of the IL-17 family of cytokines would be of interest given the potential dichotomous roles of IL-17A and IL-17E in the progression and resolution of periodontitis [27]. 

Our results, showing that LL-37 significantly increased growth factor production by gingival fibroblasts, serves to emphasise the important contribution of LL-37 to the healing response. LL-37 treatment of gingival fibroblasts led to a dose-dependent increase in bFGF expression. Interestingly, topical application of recombinant bFGF for periodontal treatment has been the subject of a clinical trial in which enhanced regeneration of periodontal tissues, as deduced by percentage bone fill, was reported [28]. Our experimental observations, together with the well-described angiogenic role for LL-37 in the oral cavity [29], point towards LL-37 as a key mediator of bFGF’s role in tissue remodeling and, indeed, regeneration. With regard to HGF, it is known that this growth factor has a multitude of functions including regeneration and wound healing of many tissues [30]. HGF is produced by gingival fibroblasts in response to *Porphyromonas gingivalis* LPS and in response to other bacterial pathogen-associated molecular patterns (PAMPs) [31,32]; in this study we now show that HGF is produced in response to LL-37. Concentrations of HGF at periodontitis sites have been reported to be almost twice that of healthy sites [33], whilst HGF concentrations in GCF are approximately 8 times higher than serum concentrations, suggesting that much of the HGF present in GCF is produced by gingival fibroblasts [33]. Indeed, the increased rate of intra-oral wound healing has been attributed to increased expression of HGF and KGF by oral fibroblasts, when compared to skin fibroblasts [34]. Furthermore, we have previously shown that oral fibroblasts produce significantly higher levels of both HGF and KGF than their dermal counterparts [35]. Previously, the expression of hCAP18 has been shown to be induced during re-epithelialisation of organ-cultured skin wounds and antibodies against LL-37 inhibited re-epithelialisation in a concentration-dependent fashion [3]. The importance of LL-37 in stimulating keratinocyte migration and wound resolution has been described in an elegant series of in vitro and in vivo experiments [36]. The stimulatory effect of LL-37 on KGF secretion by fibroblasts in the current study may suggest a role for LL-37 in keratinocyte recruitment to the wound site. 

LL-37’s potential role in the remodeling and maintenance of oral tissues is not yet fully understood but in the current study, treatment of oral fibroblasts with LL-37 resulted in no significant effect on fibroblast proliferation, as determined by MTT assay. Previous studies have reported a complex relationship between LL-37, proliferation, and MMP activity. In studies of carcinogenesis, for example, LL-37 has been shown to elicit contrasting effects, inhibiting proliferation in the case of gastric tissues but promoting proliferation via MMP-2 expression in ovarian tissues [37,38,39,40]. With reference to tissue remodeling studies, in a rat gastric ulcer model, the cathelicidin rCRAMP was shown to increase epithelial cell proliferation via an MMP-dependent pathway [41], proposing a relationship between proliferation and MMP expression. In the current study, however, no significant changes in either fibroblast proliferation or MMP activities were observed following LL-37 treatment, suggesting that whilst the relationship between proliferation and MMP activities may hold true, LL-37 does not appear to promote either in gingival fibroblasts. In the context of chronic periodontitis, where LL-37 expression has been shown to be significantly higher than in healthy sites [6], the lack of a stimulatory effect by LL-37 on MMP activities, in addition to the increased expression of TIMP-1 by the fibroblasts, may be interpreted as a refined mechanism of controlling proteolytic activity in a bid to curb disease progression. 

LL-37 has previously been shown by us to be present in periodontitis sites, despite the fact that it is susceptible to degradation at sites that are positive for *P. gingivalis* [6]. We hypothesised that LL-37 may have a role in regulating the remodeling of periodontal tissues. IL-6 and IL-8 have previously been reported in human gingival tissues in periodontitis patients [42] and increased mRNA expression of TIMP-1 has been reported in gingival tissue from periodontitis patients following nonsurgical periodontal treatment [43]. Our in vitro studies provide evidence of a role for LL-37 in modulating cytokine, growth factor, and TIMP levels. 

Local (subgingival) treatment of periodontal tissue with LL-37 or peptide mimetics of LL-37 are potential therapeutic options for periodontal disease. Both pro- and anti-inflammatory functions have previously been assigned to LL-37, depending on the microenvironment and the disease process [44], and, indeed, LL-37 has been shown to be associated with the pathology of inflammatory skin diseases such as psoriasis [45]. Any potential treatment with LL-37 should take into account our findings that LL-37 can directly increase pro-inflammatory cytokine production by gingival fibroblasts. However, given that periodontal disease is closely associated with Gram-negative anaerobes, periodontal pockets will contain abundant LPS. In such a microenvironment, LL-37 will block LPS signaling [46], which should help dampen local inflammation. Our results show that growth factor production is increased following LL-37 treatment in gingival fibroblasts, which suggests that the peptide may play an important role in periodontal tissue remodeling. 

## 5. Conclusions

In summary, treatment of gingival fibroblasts with exogenous LL-37 caused an upregulation in the production of the two pro-inflammatory cytokines tested—IL-8 and IL-6—but did not significantly affect the MMP activity profiles of the cells. However, the concentrations of TIMP-1 and various growth factors secreted by the fibroblasts (bFGF, HGF, KGF) were significantly increased following LL-37 treatment. Taken together, these results suggest an important role for the host defence peptide, LL-37, in modulating the secretion of mediators of inflammation and tissue remodeling from fibroblast cells in gingival tissues.
